# Effects of Exogenous Gibberellic Acid (GA_3_) on Nitrogen Contents and Electrophysiological Parameters in Soybean (*Glycine max* (Linn.) Merr.) Under Drought Conditions

**DOI:** 10.3390/plants15081252

**Published:** 2026-04-18

**Authors:** Deke Xing, Junle Li, Huiwen Chen, Yanyou Wu, Hai Liu, Meiqing Li, Weixu Wang

**Affiliations:** 1 Plant Stress Physiology and Ecology Laboratory, School of Agricultural Engineering, Jiangsu University, Zhenjiang 212013, China; xingdeke@ujs.edu.cn (D.X.); 19115615685@163.com (J.L.); chw2373511052@163.com (H.C.); lmqljy@163.com (M.L.); wangweixu2022@163.com (W.W.); 2 Research Center for Environmental Bio-Science and Technology, State Key Laboratory of Environmental Geochemistry, Institute of Geochemistry, Chinese Academy of Sciences, Guiyang 550081, China; 3 Institute of Crop Germplasm Resources, Guizhou Academy of Agricultural Sciences, Guiyang 550006, China; lilylike0915@163.com

**Keywords:** nitrogen accumulation, cellular metabolic electronic energy, photosynthesis, water status, seed yield

## Abstract

Exogenous application of plant hormones has been considered a short-term and effective strategy to alleviate deleterious effects of water stress on plants. However, whether exogenous gibberellic acid (GA_3_) directly enhances nitrogen accumulation and thereby alleviates drought stress in soybean (*Glycine max *(Linn.) Merr.) remains to be investigated. This study set three water treatments (75% CK, 50% MD, 25% SD), with half of the plants at each level sprayed with 10^−6^ mol·L^−1^ GA_3_, measuring growth, photosynthesis, nitrogen content, water status, and electrophysiological parameters and calculating cellular metabolic electronic energy (ΔG_B_) based on Nernst equation. The results showed that drought reduced soybean nitrogen accumulation, photosynthesis, growth and yield. GA_3_ increased soybean nitrogen accumulation, improving photosynthesis and yield under CK, which enhanced the consumption of intracellular stored energy and reduced ΔG_B_. Under MD, GA_3_ improved leaf water status, promoted soybean nitrogen accumulation and photosynthesis and reduced ΔG_B_ by allocating more energy to drought resistance; it could therefore mitigate the moderate drought stress on plants. ΔG_B_ negatively correlated with total nitrogen content and yield, indicating that ΔG_B_ was a potential indicator associated with nitrogen accumulation, which can guide the optimization of GA_3_ spraying strategies. Further studies on GA_3_ application details are necessary to improve the soybean yields under drought conditions.

## 1. Introduction

A high frequency and severity of droughts have been predicted throughout the world in the future, including most parts of China, due to global warming and the expected frequency of extreme climatic events [1]. Drought may cause severe reduction in crop yield when it is beyond the ability of crop to acclimatize or recover. Soybean (*Glycine max* (Linn.) Merr.) is one of the most important vegetable protein sources globally, contributing to the agricultural economies of many countries [2]. However, soybean plants have underdeveloped root systems and require a large amount of water. Among leguminous crops, they are the most sensitive to water. Accordingly, the annual yield loss of soybean caused by drought is enormous [3]. In view of this, considerable efforts have been made to enhance drought tolerance in soybean plants, with the primary goal being to enhance yield under drought conditions, which is of great significance for the sustainable development of soybean production [4].

Drought is the main restricting agent for the growth and development of soybean plants [5]. Remarkably, the exploitation of plant growth regulating phytohormones for decreasing the adverse effect of abiotic stress has acquired much attention in recent years. The exogenous application of plant growth regulators has been considered a short-term and effective strategy to alleviate the deleterious effects of water stress on plants [6]. Agboma et al. [7] have reported that exogenous application of glycinebetaine (GB) considerably improved the growth and yield attributes in soybean plants under water stress conditions. Salicylic acid (SA)-induced drought stress acclimation is closely related to reactive oxygen species (ROS) metabolism [8]. Brassinosteroids (BR) can enhance the protective enzyme system in plants and strengthen the ability to scavenge reactive oxygen free radicals, thereby mitigating the damage of drought to soybean plants [9]. It has been revealed that modifying the endogenous plant hormone content and antioxidant enzymes by melatonin application improved drought tolerance in soybean seedlings. Exogenous application of abscisic acid (ABA), gibberellic acid (GA_3_) and jasmonic acid (JA) can improve soybean plant’s antioxidant capability and drought tolerance [10,11], while GA_3_ exhibits a more prominent drought-mitigating effect than IAA and 6-BA in soybean seedlings [12].

Studies have also shown that application of gibberellin (GA) can enhance the drought resistance of wild soybean seedlings [12]. Khan et al. [13] described that the inoculation of LH02 in soybean plant improved its stress tolerance by inducing GA and secondary metabolite production. Gibberellin (GA) is one of the common plant hormones; its main function is to promote seed germination, elongation and division of plant cells, plant flowering, and fruit development, etc., and GA is widely used in agricultural production [14]. Interestingly, research has shown that GA can promote the formation of nitrogen-fixing organs [15] and affect the establishment of functional nodules and the formation of infection lines [16]. Nitrogen fixation in the nodules is an important way to reduce nitrogen in the earth’s ecosystem to ammonia that can be absorbed and utilized by plants [17]. When soybean plants suffer from drought, restoring or promoting symbiotic nitrogen fixation can effectively promote nitrogen accumulation in plants, reduce the dependence on nitrogen fertilizer during soybean planting, and diminish the occurrence of environmental problems caused by the excessive application of nitrogen fertilizer. However, the effect of GA_3_ on soybean is highly complex. Exogenous application of GA_3_ to soybean roots induces the expression of key nodulation genes and alters the infection thread and nodulation phenotypes, ultimately leading to differences in the size, number, and morphology of soybean nodules [18]. GA_3_ exerts complex stage-dependent effects during nodulation, rather than simple and consistent positive effects. It is still uncertain whether external GA can directly promote the nitrogen accumulation of soybean plants under drought; the mitigating effect of GA_3_ on drought stress in soybean under varying drought levels also remains unclear.

Nitrogen is considered the primary element limiting plant growth and production [19]. Plants differ in nitrogen demands and acquisition, which depend on the specific environmental conditions and biological adaptation strategies. Soybeans are nitrogen-fixing plants that can acquire nitrate nitrogen from the soil, ammonium nitrogen through symbiotic nitrogen fixation, or both types of nitrogen through artificial fertilization [20]. The majority of ammonium nitrogen is translocated to reproductive organs, including flowers and pods. However, a certain portion is also transported to leaves, as reported by Ribeiro et al. [21]. A moderate increase in nitrogen accumulation effectively enhances plant drought resistance; however, this effect is dose- and morphology-dependent and does not follow a simple linear pattern [22]. Plant leaf cells possess ammonium and nitrate nitrogen transport systems. At low nutrient concentrations, a high-affinity transport mechanism is employed, whereas a low-affinity transport mechanism is utilized at high nutrient concentrations. Both the transport and utilization of NH_4_^+^ and NO_3_^-^ necessitate energy expenditure [23]. An increase in nitrogen fixation within plants will enhance the utilization of ammonium, which has the potential to promote nitrogen accumulation within plant tissues [24]. Consequently, the metabolic energy demand varies depending on the affinity system in operation and the nitrogen accumulation [25].

Cellular metabolic electronic energy (ΔG_B_), along with chemical energy (e.g., ATP), collectively forms energy stored within cells, which is consumed by organisms to directly construct their structures or maintain life-sustaining activities [26]. Measuring changes in intracellular stored energy can directly and promptly reflect the nitrogen utilization characteristics of plants. Currently, the energy charge status within cells is mainly used to reflect the intracellular stored energy in organisms [27]. However, in reality, the demand and supply of metabolic energy for the assimilation and dissimilation of many substances remain unclear, and the same applies to numerous metabolic processes. Simply measuring the energy charge status of cells cannot truly represent the intracellular stored energy within plants. Even if the intracellular energy charge status can reflect the intracellular stored energy in organisms, it requires in vivo intracellular energy charge status, which is also difficult to achieve with existing technologies [28]. Therefore, this study will apply electrophysiological techniques to rapidly and non-destructively detect ΔG_B_ in plant leaves. Plant electrophysiology has been widely used in the study of plant water metabolism, which is attributed to its simple measurement and sensitive response [29,30]. A single leaf cell can be approximately regarded as a concentric spherical capacitor [31]. The electrolyte solution on both sides of cell membrane is regarded as two pole plates of the capacitor, the cell membrane is the medium between the pole plates, and various types of organelles in the cell are equivalent to the resistor. Many aligned cells make up the leaf capacitor by plasmodesmata. The electrophysiological parameters, i.e., capacitance, resistance and impedance, change with changing cell membrane permeability, which can be affected by the environmental changes. By measuring the electrophysiological parameters, the ΔG_B_ of a plant can be deduced and calculated according to the Gibbs free energy and Nernst equations [32,33].

In the present study, soybean plants, which suffered from drought conditions, were sprayed with GA_3_. The electrophysiological parameters, photosynthetic characteristics, leaf water status, plant and soil nitrogen contents, and plant growth were then measured. The nutrient utilization efficiency (NUUE), active nutrient transfer ability (NAT), passive transfer ability (NPT), intracellular water-use efficiency (IWUE) and ΔG_B_ of plants were calculated. We hypothesize that the exogenous application of GA_3_ can enhance the nitrogen accumulation of soybean plants under drought conditions and thereby alleviate drought stress in soybean plants. This study aims to investigate the relationship between exogenous GA_3_ application and total nitrogen content, photosynthesis and yields in soybeans under drought stress, analyzing the correlation between the electrophysiological parameters and plant nitrogen accumulation. It also provides a theoretical basis for studying the dynamic characteristics of plant total nitrogen content by using electrophysiological parameters, which is of great significance for quickly optimizing GA_3_ spraying strategy and fertilizer conservation.

## 2. Results

### 2.1. Effects of Spraying GA_3_ on Leaf Water Potential (Ψ_L_) and Leaf Water Content (LWC) Under Drought

In the absence of GA_3_ spraying, the Ψ_L_ and LWC values exhibited a notable and consistent decline, as the severity of drought stress escalated (Table 1). The application of GA_3_ significantly elevated the Ψ_L_ values of plants under the MD treatment level. Additionally, it led to an increase in the LWC values of plants in both the CK and MD treatment groups. However, the application of GA_3_ did not exert a significant influence on the Ψ_L_ values of plants under both the CK and SD treatments. In the SD treatment group, the spraying of GA_3_ failed to induce any significant change in LWC as well.

According to two-way ANOVA, drought stress had a highly significant effect on both Ψ_L_ and LWC, GA_3_ application had a significant effect on Ψ_L_ and LWC, and the interaction between drought and GA_3_ was not significant for either parameter.

### 2.2. Effects of Spraying GA_3_ on Electrophysiological Parameters Under Drought

In the absence of GA_3_ spraying, the ΔG_B_ values decreased remarkably, as the drought stress increased (Table 2). The NAT value under MD treatment level showed no clear difference with that under the SD level but was remarkably higher than that under CK treatment level. The NPT value significantly increased under the SD level compared to the values under CK and MD treatments. However, the IWUE value significantly decreased under the SD level compared to those under CK and MD treatments. The NUUE value under CK treatment level was clearly higher than that under the SD level.

The application of GA_3_ on plants significantly reduced the ΔG_B_ values at each treatment level, and those ΔG_B_ values of plants at all the treatment groups had no clear difference after spraying with GA_3_. Plants applied with GA_3_ clearly improved the NAT value under the CK treatment level, and the NPT value decreased under the SD treatment level. The IWUE value under the SD treatment level was remarkably increased by the application of GA_3_ on plants. Spraying GA_3_ had no significant effect on the NUUE of plants at every treatment level.

According to two-way ANOVA, drought stress, GA_3_ application, and their interaction all had highly significant effects on ΔG_B_. For NAT, drought stress had a highly significant effect, while GA_3_ application and their interaction were not significant. For NPT, drought stress, GA_3_ application, and their interaction all had highly significant effects. For IWUE, drought stress and the interaction between drought and GA_3_ had highly significant effects, and GA_3_ application had a significant effect. For NUUE, drought stress had a significant effect, while GA_3_ application and their interaction were not significant.

### 2.3. Effects of Spraying GA_3_ on Nitrogen Contents in Plants and Soil Under Drought

In this experiment, soybean stems, leaves and pods were defined as aboveground parts. In the absence of GA_3_ spraying, the aboveground nitrogen contents under MD and SD treatments exhibited no clear difference but were all significantly lower than that under the CK treatment level. The nitrogen content of the aboveground parts in the GA_3_-treated group increased by 14.03% compared with the CK group without GA_3_ spraying. Similarly, in the MD treatment group, the aboveground nitrogen content exhibited a 19.44% increase when GA_3_ was sprayed, as opposed to the plants without GA_3_ application. The application of GA_3_ on plants had no remarkable effect on the aboveground nitrogen content of plants under SD treatment.

The soybean roots and the nodules attached to roots were defined as the underground part. The plants without GA_3_ spraying showed a significant decrease in the nitrogen contents of the underground part with increasing drought stress levels. Compared with the plants without GA_3_ spraying, the underground part nitrogen content under the CK treatment level increased by 32.84%, and that under the SD level increased by 20.62% after GA_3_ application. The application of GA_3_ on plants had no remarkable effect on the underground nitrogen content of plants under MD treatment.

The total amounts of nitrogen of the aboveground and underground parts of soybean were defined as the total nitrogen content. The plants without spraying GA_3_ also exhibited a significant decrease in the total nitrogen contents, as the drought stress increased. The application of GA_3_ on plants clearly improved the total nitrogen content at each treatment level as opposed to the plants without GA_3_ application, respectively. By spraying GA_3_, the total nitrogen contents increased by 18.04%, 14.34% and 7.76% under the CK, MD, SD treatments, respectively.

The soil nitrogen content before planting soybean was determined as a comparison to calculate the changes in soil nitrogen content (nitrogen absorbed form soil) at every drought level. In this experiment, the nitrogen contents absorbed form soil were independent of the drought stress levels; spraying GA_3_ also showed no significant effect on the values.

According to two-way ANOVA, drought stress and GA_3_ application both had highly significant effects on the nitrogen content of the aboveground part of the plant, the underground part of the plant, and the total content in the plant, while the interaction between drought and GA_3_ had a significant effect on the nitrogen content of the aboveground part of the plant and highly significant effects on the underground part of the plant and the total content in the plant. The nitrogen content absorbed from soil, drought stress, GA_3_ application, and their interaction were all not significant (Table 3).

### 2.4. Effects of Spraying GA_3_ on Photosynthetic Characteristics Under Drought

**Figure 1:**
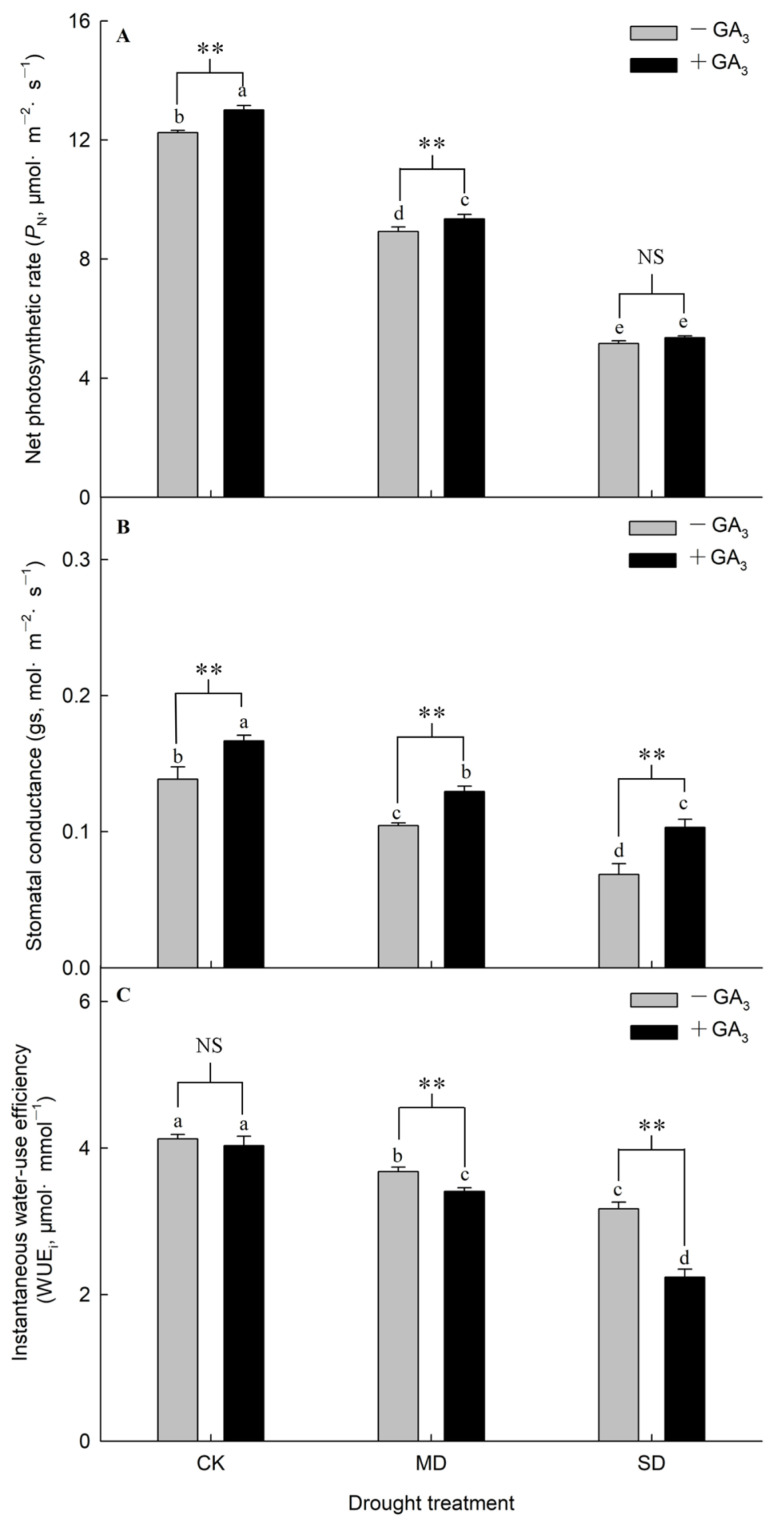
Effects of spraying GA_3_ on net photosynthetic rate, stomatal conductance and instantaneous water-use efficiency under drought (Note: (**A**), *P*_N_; (**B**), gs; (**C**), WUE_i_; +GA_3_ represents spraying with GA_3_; −GA_3_ represents not spraying GA_3_; CK represents control, MD represents moderate drought, and SD represents severe drought; different letters appear above the error bars of the same parameter when subsequent values differ significantly at *p* ≤ 0.05, according to one-way ANOVA, *n* = 5). Based on two-factor ANOVA, ** denotes a highly significant difference (*p* < 0.01), and NS indicates no significant difference.

In the absence of GA_3_ spraying, the *P*_N_, gs and WUE_i_ of plants significantly decreased with increasing drought stress levels. Spraying GA_3_ significantly increased the *P*_N_ values under the CK and MD treatments. Compared with the plants without GA_3_ spraying, the *P*_N_ of plants under the CK and MD treatments increased by 9.84% and 8.38% after GA_3_ application, respectively. The application of GA_3_ on plants had no remarkable effect on the *P*_N_ under SD treatment (Figure 1A). The gs of plants significantly increased after GA_3_ application, as opposed to the plants without GA_3_ application at each treatment level. After the application of GA_3_, the WUE_i_ of plants under the CK treatment remained stable, while the values under MD and SD treatments significantly decreased compared with the plants without GA_3_ spraying, respectively (Figure 1B,C).

### 2.5. Effects of Spraying GA_3_ on Growth Indices Under Drought

The plant heights decreased significantly with increasing drought stress levels. Spraying GA_3_ had no clear effect on plant height at each treatment level, but the plant height under the MD treatment increased slightly after GA_3_ application. The leaf areas of plants without spraying GA_3_ also significantly decreased with the increase in the drought stress levels. Similarly, spraying GA_3_ also had no significant effect on the leaf area at each treatment level (Table 4).

According to two-way ANOVA, drought stress had a highly significant effect on the plant height and leaf area both at the fifth day before and after spraying GA_3_. GA_3_ application and the interaction between drought and GA_3_ were not significant for the plant height or leaf area at either time point.

**Figure 2:**
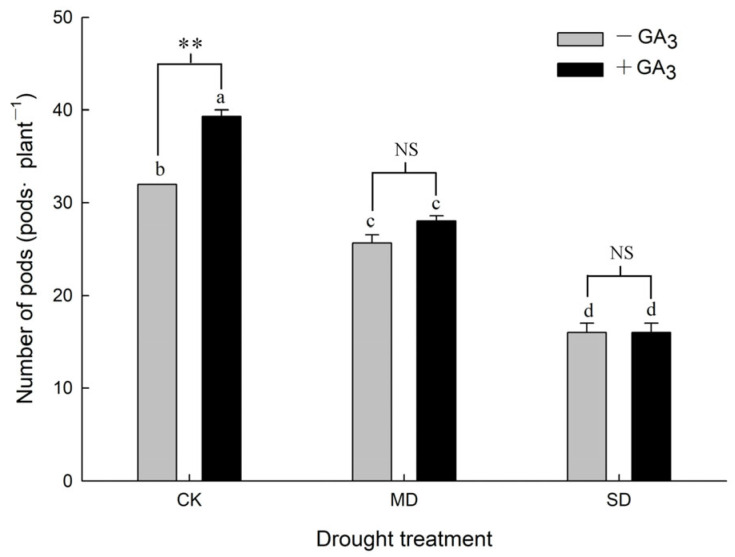
Effects of spraying GA_3_ on number of pods per plant under drought (Note: +GA_3_ represents spraying with GA_3_; −GA_3_ represents not spraying GA_3_; CK represents control, MD represents moderate drought, and SD represents severe drought; Different letters appear above the error bars of the same parameter when subsequent values differ significantly at *p* ≤ 0.05, according to one-way ANOVA, *n* = 5). Based on two-factor ANOVA, ** denotes a highly significant difference (*p* < 0.01), and NS indicates no significant difference.

In the absence of GA_3_ application, the pods decreased significantly with increasing drought stress levels; the pods under MD and SD treatments decreased to 70.26% and 40.51% of that under CK, respectively. Spraying GA_3_ significantly promoted the pods under the CK treatment, which exhibited a 21.88% increase when the GA_3_ was sprayed. Spraying GA_3_ had no clear effect on the pods of plants under the MD and SD treatments (Figure 2).

### 2.6. Correlation Analysis Discussion

The total nitrogen content was extremely negatively correlated with ΔG_B_ and extremely positively correlated with the number of pods, with correlation coefficients of −0.76 and 0.92, respectively. ΔG_B_ also showed a significant negative correlation with the number of pods, with a correlation coefficient of −0.80. NUUE was significantly negatively correlated with NAT and positively correlated with IWUE, plant height, leaf area, and *P*_N_, with correlation coefficients of −0.41, 0.46, 0.38, 0.44, and 0.42, respectively. NPT was negatively correlated with IWUE, leaf area, and *P*_N_, with correlation coefficients of −0.79, −0.47, and −0.43, respectively. IWUE showed significantly extremely positive correlations with plant height, leaf area, and *P*_N_, with correlation coefficients of 0.47, 0.57, and 0.52, respectively. Additionally, extremely positive correlations were observed between plant height and leaf area (0.89), plant height and *P*_N_ (0.92) and leaf area and *P*_N_ (0.95), respectively (Table 5).

## 3. Discussion

In the current study, we used a self-made parallel plate capacitor to capture plant electrical signals in a timely and non-invasive manner to explore the dynamic traits of intracellular substances and electronic energy in soybean plants. Solute concentration, cell volume, and ion permeability depend on nutrients and water content in cells under drought stress, and they will inevitably change the dielectric constant of leaf tissue, which finally affects the electrophysiological parameters of plants, including IC, IR, IX_C_, and IXL. The IWUE is related to the cell volume and intracellular water use, the nutrient use parameters including NAT, NPT and NUUE are calculated using IR, IX_C_, and IXL, and the ΔG_B_ calculated according to the Gibbs free energy and Nernst equations reflects the cellular metabolic electronic energy [33]. Electrophysiological techniques have been successfully applied to detect the dynamics of water, nutrients and metabolic energy in plant leaf cells and to analyze plant water status [30,32,34].

### 3.1. Effects of Drought on Electrophysiological Parameters, Water Status, Nitrogen Absorption, Photosynthesis and Growth of Soybean Plants

Leaf water potential serves as a sensitive indicator of plant water status, representing the sum of all forces influencing water movement within a plant [35]. In the present study, we observed that Ψ_L_ decreased as drought stress increased and coincided with a decrease in LWC. Additionally, the leaf water status interacts with gs and E, and the results in this study showed that the gs decreased with an increasing drought stress level. Stomatal closure in plants under drought stress is a common regulatory mechanism. As a consequence, water loss by plant transpiration was reduced by the closing of stomata. Similar findings have previously been reported in soybeans [36]. The transpirational pull decreased due to the reduction in plant transpiration, which would lower the transport efficiency of nutrients through the whole plant. Interestingly, this study also found that the active (NAT) and passive transport (NPT) capacities of nutrients in leaf cells were increased under severe drought stress, which, however, could not prevent the decline in the use efficiency of water and nutrients in plant cells. As a result, a significant decrease in IWUE and NUUE was observed under severe drought stress.

The nitrogen content in plants has also been shown to decrease, as Ψ_L_ and gs decrease [37]. As observed in our study, drought decreased the nitrogen accumulation in soybean plants, especially in the underground parts. Nitrogen is a major component of chlorophyll and photosynthetic enzymes, and plants need to absorb enough nitrogen to support leaf development and photosynthetic performance [38]. The significant decrease in nitrogen accumulation exerted a negative impact on the photosynthesis of soybeans. In addition, the gradually closing stomata under drought enhanced the gas exchange resistance and remarkably reduced the CO_2_ absorbed by soybeans, ultimately leading to a significant decrease in *P*_N_ with increasing drought levels. Furthermore, through *P*_N_ and Tr, it was calculated that WUE_i_ also decreased significantly, implying the amount of carbon fixed by plants per unit of water consumed has decreased.

Photosynthesis is the most important driving force for plant growth and yield formation, because it provides the energy and organic matter required for the plant growth process [38]. Lower *P*_N_ under drought conditions decreased the production of carbohydrate in soybean plants and further impaired the capacity of plants to supply energy required for maintaining cellular metabolism under drought. According to our experiment, ΔG_B_ significantly decreased with the intensification of drought, indicating that drought conditions caused plants to consume more energy to resist stress, thereby reducing the cellular metabolic electronic energy, which is not conducive to plant life activities and growth. We also observed that drought stress inhibited the nutritional growth of soybean plants and made their overall morphology smaller. This was consistent with Fehr’s claim that drought could prolong the nutritional growth period of soybean plants, which had slower nutritional growth under drought conditions [39]. However, it is worth noting that the extension of the nutritional growth period was accompanied by a shortening of the reproductive period in soybean plants, resulting in a decrease in yield.

### 3.2. Effects of Spraying GA_3_ on Electrophysiological Parameters, Water Status, Nitrogen Absorption, Photosynthesis and Growth of Soybean Plants Under Different Drought Conditions

GA_3_ can enhance photosynthesis in soybeans and increase the biomass accumulation, which eventually improves the growth and yield. GA_3_ promotes stem elongation and cell division in plant shoot caused by the direct regulation of protein and RNA (ribonucleic acid) synthesis [14]. In the present study, positive influences of GA_3_ on the LWC, NAT, nitrogen contents in the aboveground and underground parts, *P*_N_, gs, and number of pods were clearly observed in the plants under the CK treatment. Exogenous application of GA_3_ increased the water content in leaves and enhanced the gas exchange efficiency by promoting stomatal opening. Meanwhile, the GA_3_ spraying augmented the nitrogen absorption and accumulation in soybean plants; therefore, the yield of soybeans was improved remarkably. We also observed that the GA_3_ spraying just slightly increased the plant height but had no promotion effect on the leaf area. Our results confirmed earlier observations of Rademacher [40] that gibberellins boost longitudinal growth caused by the development of meristematic tissues. The increased accumulation of nitrogen in plants and seed yields elevated the consumption of intracellular stored energy, which consequently decreased the ΔG_B_ substantially after spraying GA_3_.

Spraying GA_3_ exerted varying effects on soybean plants under drought conditions. According to two factor analysis, drought stress significantly affected all measured physiological and growth parameters, while GA_3_ application showed significant effects on most parameters except NAT, NUUE, plant height, and leaf area. In the MD treatment group, spraying GA_3_ improved the water status of soybean plants in line with the earlier findings of El-Tohamy et al. [41]. However, the foliar application of GA_3_ did not change the dynamics of the intracellular water and nutrient of soybean plants in MD treatment, which was demonstrated by the stability of the values of NAT, NPT, IWUE and NUUE before and after spraying GA_3_. It is worth noting that the improvement of the plant water status increased gs and also remarkably promoted the accumulation of nitrogen in the aboveground parts of soybeans. Although the transpiration dissipation of plants was increased, which at the same time declined the WUE_i_, the augmented nitrogen accumulation in the aboveground parts of soybeans ensured the increase in *P*_N_. The increased gs concomitantly with a reduction in WUE_i_ under drought stress could be attributed to the enhanced carbon assimilation efficiency of individual stomata during drought, while leaf-level stomatal regulation fails to counteract the overall decline in WUE_i_ caused by drought stress [42]. As we know, leaves are the largest nitrogen sinks in plants, and up to three quarters of the nitrogen that leaves accumulate is fed into the photosynthetic apparatus [43]. Therefore, the photosynthetic capacity of soybeans was promoted, which increased the generation of photosynthate in plants.

In the present study, we just observed a slight increase in plant height under MD treatment. The ΔG_B_ of plants after spraying GA_3_ became only 56.57% of the value of plants under the MD treatment. The increased accumulation of nitrogen in plants and seed yields, together with the energy used for resisting adversity, elevated the consumption of intracellular stored energy, which consequently further declined the ΔG_B_ substantially after spraying GA_3_ under MD treatment. As a result, spraying GA_3_ alleviated the negative effects of moderate drought condition on soybean plants.

Under SD conditions, spraying GA_3_ failed to improve the plant water status and had no promoting effect on the transport capacity of intracellular substances; the NPT was eventually decreased. On the contrary, the use efficiency of intracellular water and nutrients by soybean plants increased to varying degrees. However, even the remarkable increase in the gs of plants after the foliar application of GA_3_ could not promote photosynthesis, which increased the transpiration dissipation instead and ultimately decreased the WUE_i_. Interestingly, spraying GA_3_ also increased the nitrogen accumulation in the underground parts of soybean plants, but the absorbed nitrogen could not be effectively transferred to the aboveground parts. In this situation, soybean plants suffered from serious damage; energy converted from the decomposition of photosynthates was mainly used to resist drought stress, which reduced the energy supply for cellular metabolic activities and plant growth. Therefore, the foliar application of GA_3_ had no positive effects on the *P*_N_, growth and seed yield under the SD treatment. Chen et al. [44] also found that the mitigating effect of gibberellin on drought-induced stress in *Festuca arundinacea* seedlings exhibited a gradual attenuation, as the intensity of drought stress increased.

**Figure 3:**
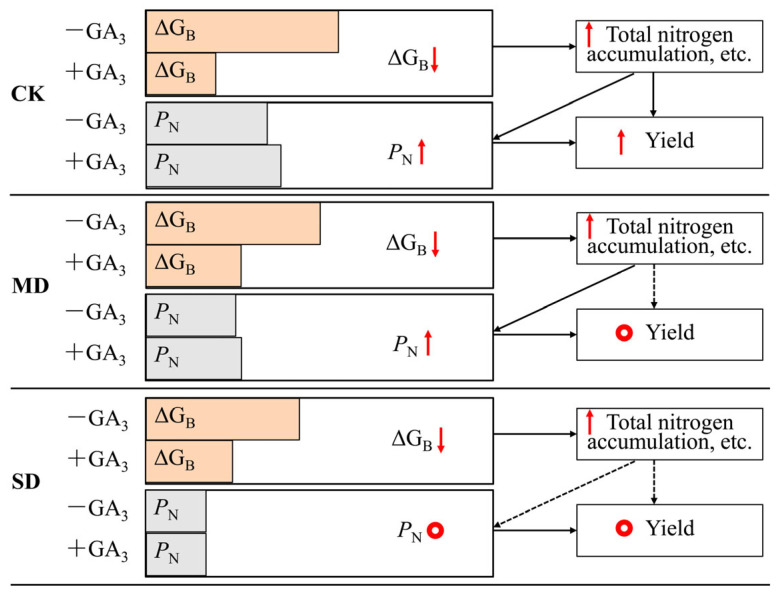
The response of ΔG_B_, *P*_N_, yield, and total nitrogen contents of soybean to drought. Note: 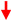 represents decrease; 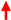 represents increase; 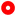 represents stable.

Based on the comprehensive analysis of ΔG_B_, nitrogen accumulation, yield, and photosynthetic performance, we proposed a conceptual model (Figure 3) to illustrate the effects of GA_3_ on ΔG_B_, *P*_N_, number of pods (as yield), and total nitrogen accumulation of soybean under different drought levels. After light energy conversion, it was stored in cells in the forms of electronic energy (ΔG_B_) and chemical energy. With the intensification of drought stress, the total amount of electronic energy and chemical energy produced by light energy conversion decreased, and ΔG_B_ decreased. After spraying GA_3_ under each treatment, the accumulation of nitrogen in plants increased significantly, a process that enhanced the consumption of energy stored in cells. The energy consumption caused by nitrogen accumulation reduced the remaining electronic energy in cells; therefore, after GA_3_ spraying, ΔG_B_ under each treatment further decreased significantly. The increase in nitrogen accumulation was conducive to the improvement in photosynthetic capacity and also promoted the yield to varying degrees, with the magnitude of the yield increase affected by the degree of drought [45].

In the present study, the total plant nitrogen content was significantly increased by GA_3_ application among all drought levels, whereas the soil total nitrogen showed no significant difference before and after the experiment. Based on these observations, enhanced biological nitrogen fixation could be regarded as one possible explanation for the higher plant nitrogen accumulation. However, this was by no means the only interpretation. Multiple alternative mechanisms may also contribute to the observed changes, i.e., altered nitrogen remobilization from old leaves to young tissues, changed root nitrogen uptake kinetics or efficiency, modified root system architecture that promotes soil nitrogen acquisition, biomass dilution effects caused by accelerated growth, and shifts in biomass partitioning between shoots and roots. Since none of these processes were directly measured in this experiment, no single mechanism can be confirmed. The increased total nitrogen in plants should be regarded as the comprehensive result of multiple potential physiological changes. Further studies combining nodule phenotyping, nitrogenase activity and molecular detection are needed to reveal the specific relationship between ΔG_B_ and nitrogen fixation.

### 3.3. Correlation Analysis

The ΔG_B_ was negatively correlated with the total nitrogen content and number of pods. Meanwhile, the results of this study indicated that foliar GA_3_ application increased the total nitrogen content of soybean plants across all treatment levels and simultaneously reduced the ΔG_B_. At each drought stress level, increased nitrogen accumulation and seed yields in plants after spraying GA_3_ elevated the consumption of intracellular stored energy; plants would also allocate partial energy for resisting drought stress. As a result, the ΔG_B_ value showed a significant decrease. However, this process was conducive to alleviating the negative effects of moderate drought conditions on soybean plants. Therefore, the ΔG_B_ was strongly correlated with the total nitrogen content of soybean plants and might characterize the dynamic characteristics of nitrogen accumulation in soybean plants following further experimental validation; this electrophysiological parameter can therefore be used for the rapid and non-destructive assessment of nitrogen utilization traits in soybean plants.

## 4. Materials and Methods

### 4.1. Plant Materials and Treatment

The experiment was carried out in the greenhouse of Jiangsu University (32°11′ N, 119°25′ E). Zhonghuang 13 soybean was used as the experimental material. Before planting, the seeds were disinfected with 0.3% potassium permanganate solution and then soaked in clean water for 2 h. After soaking, the seeds were sown in a soil depth of 3~5 cm in the plastic pots. Here, 12 kg of completely dried clay soil was loaded into each plastic pot; the soil had 34.88% field capacity and 1.17 g/cm^3^ bulk density. Each pot was watered at the same time every day until the seedlings emerged. Then, 30 uniformly grown soybean seedlings (one seedling per pot) were randomly selected for the following treatment. Three levels of drought treatments were set up and maintained by a weighing method, including normal water supply (the soil relative water content is 75% ± 5%, CK), moderate drought (the soil relative water content is 50% ± 5%, MD) and severe drought (the soil relative water content is 25% ± 5%, SD). Firstly, the soil volume in each pot was calculated according to the soil weight and bulk density. The soil water content was the product of the soil relative water content (SWC_R_) and field capacity; the water addition for each SWC_R_ level was the product of the soil volume in each pot and the soil water content. During the drought treatment period, water supplementation was carried out daily at 16:00~17:00 to maintain the soil moisture of each drought treatment level, and the drought treatment lasted for 40 days.

Under each drought treatment level, half of the seedlings (5 plants as 5 replicates) were sprayed with GA, while the remaining seedlings (also 5 plants as 5 replicates) had no GA spraying. There are many types of gibberellins, including GA_1_, GA_2_, GA_3_ and so on, among which exogenous GA_3_ treatment can induce the expression of key genes in soybean nodules [18], which is the reason for choosing GA_3_ in this experiment. Because both low and high concentrations of GA_3_ signal can reduce the number of nodules and affect nitrogen fixation [15], 10^−6^ mol·L^−1^ GA_3_ was prepared and applied in this experiment. GA_3_ spraying was performed quantitatively at 9:00 a.m. every day from day 30 to day 32 after sowing, for a total of 3 consecutive days. The spraying site was precisely targeted at the junction of the root and stem, true leaves, and the apex of the main stem of each soybean plant to ensure the direct absorption of GA_3_ by the target tissues. To avoid runoff of the GA_3_ solution, the spraying was performed with a fine sprayer at a low flow rate, and each plant was sprayed evenly in a circular manner around the target, ensuring that the solution formed a thin film on the surface of the tissues without dripping. To ensure dose uniformity among different plants, the same operator performed all spraying operations, and the sprayer was calibrated before each use to ensure a consistent flow rate; additionally, the plants were arranged uniformly in the greenhouse, and the spraying order was randomized to avoid systematic errors caused by the spraying sequence. The soil nitrogen contents were determined before soybean planting and at the end of the drought treatment. The plant height and leaf area were recorded on the 5th day before spraying GA_3_ and the 5th day after the final spray. The leaf water potential (Ψ_L_), leaf water content (LWC), electrophysiological and photosynthetic parameters, and number of pods was determined at the end of the drought treatment.

### 4.2. Determination of Leaf Water Potential (Ψ_L_) and Leaf Water Content (LWC)

The leaf water potential (Ψ_L,_ MPa) was measured by using a dew point water potential meter (C-52-SF Psypro, Wescor, Logan, UT, USA). The 4th and 5th fully expanded leaves from top to bottom were randomly selected. Ten leaves (5 from GA-sprayed seedlings and 5 from seedlings without GA spraying) were used for the determination at each drought treatment level. The fresh weight (FW, g) of the leaf was weighed firstly; then, the leaf was put into an oven and roasted at 60 °C to constant weight, and finally, the dry weight (DW, g) was recorded. The leaf water content (LWC, %) was calculated as follows:

(1) $$LWC=\frac{FW-DW}{FW}\times 100\%$$

### 4.3. Determination of Electrophysiological Parameters

The electrophysiological parameters were measured using an LCR instrument (6100, GW Insteck, Taiwan) connected with a self-made parallel plate capacitor [33]. Before measurement, the electrophysiological instrument was calibrated. All measurements were performed under stable environmental conditions (uniform light intensity, consistent temperature and humidity) to avoid external interference. To minimize operator bias, all measurements were conducted by the same trained operator. To reduce inter-leaf variability, 10 leaves (5 from GA-sprayed seedlings and 5 from seedlings without GA spraying) were used for the determination at each drought treatment level. Each leaf was measured 3 times at 3 different positions; then, the average value was calculated. The 4th and 5th fully expanded leaves from top to bottom were randomly selected. The measured voltage is 1.5 V, and the frequency is 3 KHz. The C (pF), R (Ω) and Z (Ω) values of leaves under different gripping forces were recorded, and the ΔG_B_, NAT, NPT, NUUE and IWUE were calculated, respectively.

The cell sap solute in plant leaf is taken as the dielectric. The leaf is clipped between the two electrodes of the self-made parallel plate capacitor. C at different gripping forces can be determined by changing the iron mass above the electrodes of the capacitor. The cytosol solute concentration and elasticity and plasticity of the cells change as the pressure varies, which causes dielectric constant variation in the cytosol solute and C between the two electrodes of the parallel-plate capacitor.

The gripping force is calculated according to the following formula:

(2) F=(M+m)g

where F is the gripping force, and the unit is N, M is the weight of iron, and the unit is kg, m is the weight of the plastic rod and electrode sheet, and the unit is kg, g is the gravitational acceleration, and the value is 9.8 N·g^−1^.

Water content within a plant cell is correlated to the cell elasticity, and the C at a specific gripping force differs among different plants.

The Gibbs free energy equation is ΔG=ΔH+PV, the energy expression of the capacitors is $W=\frac{1}{2}U^{2}C$, and W is the energy of the capacitor, which is equal to the work converted from Gibbs free energy; that is, W=ΔG. ΔH is the internal energy of the system (the plant leaf system which composed of cells). V is the volume of the leaf cell; U is the test voltage. P is the pressure on plant cells, $P=\frac{F}{S}$, S is the effective area of leaf that is in contact with capacitor, and F is the gripping force. The change in C of plant leaves with F can be expressed as follows:

(3) $$C=\frac{2\Delta H}{U^{2}}+\frac{2V}{SU^{2}}F$$

d represents the specific effective thickness of plant leaves; $d=\frac{V}{S}$, and Equation (3) was transformed into

(4) $$C=\frac{2\Delta H}{U^{2}}+\frac{2d}{U^{2}}F$$

Incorporating $x_{0}=\frac{2\Delta H}{U^{2}}$ and $h=\frac{2d}{U^{2}}$ into Equation (4), changes this equation to

(5) $$C=x_{0}+hF$$

Since $h=\frac{2d}{U^{2}}$, d can be calculated as $d=\frac{U^{2}\times h}{2}$.

The leaf resistance values change with the changing cell water content and cell membrane permeability. The latter can be influenced by the external stimulus, which changes the ion concentrations inside and outside of the membrane. The Nernst equation can be used to describe the changes in the concentration of ions, ion groups and electric dipoles inside and outside of the cell membrane. The R value depends on the concentration of ions inside and outside the membrane and follows the Nernst equation, which is expressed as follows:

(6) $$E-E^{0}=\frac{R_{0}T}{n_{R}F_{0}}ln\frac{C_{i}}{C_{o}}$$

where E: the electromotive force (V), E^0^: the standard electromotive force (V), R_0_: the gas constant (8.31 J·K^−1^·mol^−1^), T: the thermodynamic temperature (K), C_i_: the concentration of electrolytes that respond to R inside the cell membrane (mol·L^−1^), C_o_: the concentration of electrolytes that respond to R outside the cell membrane (mol·L^−1^), F_0_: faraday constant (9.65 × 10^4^ C·mol^−1^), and n_R_: the number of transferred electrolytes (mol).

The internal energy of the electromotive force can be converted into pressure work, and they have a direct relationship, PV = aE; that is,

(7) $$PV=aE=aE^{0}+\frac{aR_{0}T}{n_{R}F_{0}}ln\frac{C_{i}}{C_{o}}$$

where P is the pressure on the cell, a is the transfer coefficient from electromotive force to energy, and V is the cell volume.

In terms of the mesophyll cell, the sum of C_o_ and C_i_ is constant, which is equal to the total ion concentration inside and outside of the membrane, C_T_ is the total ion concentration in response to R inside and outside the cell, $C_{T}=C_{i}+C_{o}$. C_i_ is positively correlated to electrical conductivity, and the electrical conductivity is the reciprocal of R. Therefore, $\frac{C_{i}}{C_{o}}$ can be expressed as

(8) $$\frac{C_{i}}{C_{o}}=\frac{\frac{f_{0}}{R}}{C_{T}-\frac{f_{0}}{R}}=\frac{f_{0}}{C_{T}R-f_{0}}$$

where f_0_ is the transfer coefficient between C_i_ and R; so, we obtain

(9) $$\frac{V}{S}F=aE^{0}-\frac{aR_{0}T}{n_{R}F_{0}}ln\frac{C_{T}R-f_{0}}{f_{0}}$$

Equation (9) can be transformed into

(10) $$\frac{aR_{0}T}{n_{R}F_{0}}ln\frac{C_{T}R-f_{0}}{f_{0}}=aE^{0}-\frac{V}{S}F$$

Further,

(11) $$ln\frac{C_{T}R-f_{0}}{f_{0}}=\frac{n_{R}F_{0}E^{0}}{R_{0}T}-\frac{Vn_{R}F_{0}}{SaR_{0}T}F$$

Incorporating $\alpha =\frac{n_{R}F_{0}E^{0}}{R_{0}T}\;\mathrm{and}\;\beta =\frac{Vn_{R}F_{0}}{SaR_{0}T}$ into Equation (11), changes this equation to

(12) $$ln\frac{C_{T}R-f_{0}}{f_{0}}=\alpha -\beta F$$

Taking the index on both sides of Equation (12), we have

(13) $$\frac{C_{T}R-f_{0}}{f_{0}}=e^{\alpha }e^{-\beta F}$$

Then,

(14) $$R=\frac{f_{0}}{C_{T}}+\frac{f_{0}}{C_{T}}e^{\alpha }e^{-\beta F}$$

Incorporating $y_{1}=\frac{f_{0}}{C_{T}},k_{1}=\frac{f_{0}}{C_{T}}e^{\alpha }\;\mathrm{and}\;b_{1}=\beta$ into Equation (14) changes this equation to

(15) $$R=y_{1}+k_{1}e^{-b_{1}F}$$

where $y_{1}$, $k_{1}$, and $b_{1}$ are model parameters.

Under standard conditions, the conversion of electromotive force internal energy into work is equivalent to cellular metabolic electronic energy; therefore, the unit cellular metabolic electronic energy of leaf based on R (ΔG_R-E_) is calculated as follows:

(16) $${\Delta G}_{R-E}=\frac{{aE}^{0}}{d}=\frac{{lnk}_{1}-{lny}_{1}}{b_{1}}$$

where d is the specific effective thickness of plant leaves.

The cellular metabolic electronic energy based on R (ΔG_R_) is calculated by $\Delta G_{R}{=\Delta G}_{R-E}\cdot d$.

Similarly, the relationship model between Z and gripping force can be expressed as follows:

(17) $$Z=y_{2}+k_{2}e^{-b_{2}F}$$

where $y_{2}$, $k_{2}$, and $b_{2}$ are model parameters.

The unit cellular metabolic electronic energy of leaf based on Z (ΔG_Z-E_) is calculated according to

(18) $${\Delta G}_{Z-E}=\frac{{aE}^{0}}{d}=\frac{{lnk}_{2}-{lny}_{2}}{b_{2}}$$

The cellular metabolic electronic energy based on Z (ΔG_Z_) is calculated by $\Delta G_{Z}{=\Delta G}_{Z-E}\cdot d$.

The ΔG_B_ can be expressed as

(19) $${\Delta G}_{B}=\frac{{\Delta G}_{R}+{\Delta G}_{Z}}{2}$$

After measuring the C, R and Z, the physiological capacitive reactance (X_C_) and the physiological inductive reactance (XL) can be calculated as follows:

(20) $$Xc=\frac{1}{2\pi fC}$$

(21) $$\frac{1}{-XL}=\frac{1}{Z}-\frac{1}{R}-\frac{1}{Xc}$$

where f is the test frequency, and π is equal to 3.1416.

In addition to being represented by (6), the Nernst equation can also be expressed as $E-E^{0}=\frac{R_{0}T}{n_{XC}F_{0}}ln\frac{Q_{i}}{Q_{o}}$ and $E-E^{0}=\frac{R_{0}T}{n_{XL}F_{0}}ln\frac{M_{i}}{M_{o}}$, where Q_i_ is the concentration of electrolytes that respond to X_C_ inside the cell membrane, Q_o_ is the concentration of electrolytes that respond to X_C_ outside the cell membrane, M_i_ is the concentration of electrolytes that respond to XL inside the cell membrane, and M_o_ is the concentration of electrolytes that respond to XL outside the cell membrane. According to the establishment of the relationship model between R and the gripping force, the relationship models between X_C_ and XL and gripping force are established:

(22) $$X_{C}=y_{3}+k_{3}e^{-b_{3}F}$$

(23) $$XL=y_{4}+k_{4}e^{-b_{4}F}$$

where $y_{3}$, $k_{3}$, $b_{3}$, $y_{4}$, $k_{4}$, and $b_{4}$ are model parameters.

When F=0, IR, IX_C_, and IXL can be calculate according to the following equations:

(24) $$IR=y_{1}+k_{1}$$

(25) $$IX_{C}=y_{3}+k_{3}$$

(26) $$IXL=y_{4}+k_{4}$$

Furthermore, the reciprocal of IX_C_ can be obtained by ${IX_{C}}^{-1}=\frac{1}{IX_{C}}$, and the reciprocal of IXL can be obtained by ${IXL}^{-1}=\frac{1}{IXL}$.

According to the reciprocal of IR, the active nutrient transfer ability (NAT) and passive transfer ability (NPT) of plant leaves based on electrophysiological parameters are calculated as follows:

(27) $$NAT=\frac{{IXL}^{-1}}{{IR}^{-1}}$$

(28) $$NPT=\frac{{IX_{C}}^{-1}}{{IR}^{-1}}$$

Finally, the plant nutrient utilization efficiency (NUUE) can be obtained as follows:

(29) $$NUUE=\frac{100}{NAT+NPT}$$

Plant cells can be seen as spherical structures, and their growth is closely related to the increase in volume. Therefore, the C of plant leaf cells can be obtained through the formula of concentric circular capacitors:

(30) $$C_{c}=\frac{4\pi \varepsilon R_{1}R_{2}}{R_{2}-R_{1}}$$

where π takes a value of 3.1416; C_c_ is the capacitance (pF) of a concentric spherical capacitor; ɛ is the dielectric constant of the electrolyte; R_1_ is the radius of the outer sphere (m); R_2_ is the inner sphere radius (m).

For the same plant cell, R_2_-R_1_ is the thickness of the cell membrane, and R_1_ ≈ R_2_. ɛ is a constant; therefore, the volume of cells (V_c_) is related to the C as follows:

(31) $$V_{c}=\alpha \sqrt{C^{3}}$$

The volume of cells is positively correlated with the volume of vacuoles, and the main components of vacuoles and cytoplasm are water; so, the water-holding capacity of cells is directly proportional to $\sqrt{C^{3}}$. Therefore, $\sqrt{C^{3}}$ can represent the water-holding capacity of plant leaves. The intracellular water-holding capacity (IWHC) of plant leaf cells can be obtained by the following equation:

(32) $$IWHC=\sqrt{({IC)}^{3}}$$

where IC is the inherent physiological capacitance, and the unit is pF. After converting the units, IC can be obtained by the formula: $IC=\frac{1}{2\times 3.1416\times 3000\times {IX}_{C}}\times 10^{6}$.

The intracellular water-use efficiency (IWUE) can be calculated as follows:

(33) $$IWUE=\frac{d}{IWHC}$$

### 4.4. Determination of Nitrogen Contents of Plant and Soil

The whole soybean plants were destructively sampled at the end of the drought treatments. They were divided into four parts, i.e., root, stem, leaf and pod. The soils were sampled before and after soybean planting. All the samples were dried and ground into powder with a Scientz-48L frozen high-throughput tissue grinder (Scientz-48L, Ningbo, China). Then, a 0.2 g sample was taken for digestion using the H_2_SO_4_·H_2_O_2_ digestion method, and N concentrations in the digestion solution were determined with an AA3 continuous-flow analyzer (AA3, Seal Analytical, Norderstedt, Germany), where the detection wavelength is set to 660 nm [46]. Finally, the nitrogen contents of the shoot and underground part, total nitrogen content, soil nitrogen contents before and after soybean planting, and the changes in soil nitrogen content were calculated. The measurement was conducted in triplicate. N (%) was calculated as follows:

(34) $$N\left(\%\right)=\frac{\rho \times n\times V_{d}}{M_{s}}\times 100\%$$

where ρ was the concentration of N in the digestion solution, n was the dilution factor, V_d_ was the volume of the digestion solution, and M_s_ was the quality of the testing sample.

### 4.5. Determination of Photosynthetic Parameters

Am LI-6400 portable photosynthetic measurement system (LI-COR, Lincoln, NE, USA) was used for the in situ measurement of photosynthetic parameters. The 4th and 5th fully expanded leaves (5 from GA-sprayed seedlings and 5 from seedlings without GA spraying) at each drought treatment level were selected for the determination. The net photosynthetic rate (*P*_N_, μmol·m^−2^·s^−1^), stomatal conductance (gs, mol·m^−2^·s^−1^) and transpiration rate (Tr, mmol·m^−2^·s^−1^) were recorded at 9:00–11:00 a.m., as described by Zhou et al. [47]. The instantaneous water-use efficiency (WUE_i_, μmol·mmol^−1^) was calculated according to the following equation:

(35) $${WUE}_{i}=\frac{P_{N}}{Tr}$$

### 4.6. Determination of Plant Height, Leaf Area and Pods

The plant height was measured from the cotyledon node to the apical meristem using a digital measuring tape with a precision of 0.1 cm. The leaf area was obtained by a scanning method using Wide Leaf Image Analysis System (WinFOLIA, Regent Instruments Inc., Quebec, QC, Canada). The number of pods was obtained by manual counting. All the measurements were conducted over five replicates.

### 4.7. Statistical Analysis

SigmaPlot 14.0 software was used for fitting curves. By using SPSS software (version 14.0, SPSS Inc., New York, NY, USA), the Shapiro–Wilk test was used to evaluate the normality of the data, and Levene’s test was used to evaluate the homogeneity of the data. Then, Duncan’s multiple comparison was used to analyze the results by one-way and two-way ANOVA at a 5% or 1% significance level (*p* ≤ 0.05 or *p* ≤ 0.01). The data are shown as the means ± SE. Origin 2019 software was used for plotting.

## 5. Conclusions

Drought stress significantly reduced the total nitrogen accumulation, photosynthate production and ΔG_B_ in soybean, thereby inhibiting plant growth and seed yield. GA_3_ application effectively increased the total nitrogen accumulation and photosynthetic capacity and decreased ΔG_B_ in well-watered conditions; under MD treatment, GA_3_ improved the leaf water status and total nitrogen accumulation, which was conducive to improve the photosynthetic capacity of plants. Enhanced nitrogen accumulation in plants increased the consumption of intracellular stored energy and reduced ΔG_B_ but alleviated the drought stress on soybean under the MD treatment. However, GA_3_ had no significant positive effects on soybean under the SD treatment. ΔG_B_ was strongly correlated with the soybean total nitrogen content, showing potential as a rapid non-destructive electrophysiological indicator for monitoring nitrogen accumulation in soybean.

## Figures and Tables

**Table 1:** Effects of spraying GA_3_ on Ψ_L_ and LWC under drought.

Treatments		Ψ_L_ (MPa)	LWC (%)
CK	−GA_3_	−2.21 ± 0.04 ab	73.57 ± 0.88 b
	+GA_3_	−2.07 ± 0.05 a	79.00 ± 1.00 a
MD	−GA_3_	−2.40 ± 0.04 c	55.71 ± 1.43 d
	+GA_3_	−2.27 ± 0.01 b	62.88 ± 2.33 c
SD	−GA_3_	−2.82 ± 0.04 d	34.67 ± 1.33 e
	+GA_3_	−2.71 ± 0.08 d	36.00 ± 1.63 e
Drought		**	**
GA_3_		*	*
Drought × GA_3_		NS	NS

Note: +GA_3_ represents spraying with GA_3_; −GA_3_ represents not spraying GA_3_. CK represents control, MD represents moderate drought, and SD represents severe drought. All the data in the table are analyzed by one-way ANOVA at *p* ≤ 0.05 and presented as mean ± SE (*n* = 5); the value in the same column is significantly different followed by different letters. Based on two-factor ANOVA, ** denotes a highly significant difference (*p* < 0.01), * denotes a significant difference (*p* < 0.05), and NS indicates no significant difference.

**Table 2:** Effects of spraying GA_3_ on electrophysiological parameters under drought.

Treatments		ΔG_B_	NAT	NPT	IWUE	NUUE
CK	−GA_3_	1947.736 ± 137.664 a	604.884 ± 5.170 b	625.898 ± 8.109 b	0.050 ± 0.002 a	0.103 ± 0.002 a
	+GA_3_	554.543 ± 52.198 d	657.348 ± 8.062 a	621.769 ± 15.241 b	0.045 ± 0.003 a	0.091 ± 0.005 ab
MD	−GA_3_	1224.780 ± 50.712 b	659.949 ± 6.463 a	626.303 ± 14.589 b	0.045 ± 0.001 a	0.089 ± 0.007 ab
	+GA_3_	692.837 ± 30.716 d	632.038 ± 13.382 ab	622.202 ± 6.589 b	0.048 ± 0.000 a	0.090 ± 0.006 ab
SD	−GA_3_	1021.892 ± 27.542 c	647.813 ± 5.506 a	733.949 ± 9.059 a	0.028 ± 0.001 b	0.074 ± 0.002 b
	+GA_3_	608.623 ± 26.512 d	630.418 ± 14.306 ab	615.296 ± 11.856 b	0.045 ± 0.003 a	0.089 ± 0.007 ab
Drought		**	**	**	**	*
GA_3_		**	NS	**	*	NS
Drought × GA_3_		**	NS	**	**	NS

Note: +GA_3_ represents spraying with GA_3_; −GA_3_ represents not spraying GA_3_. CK represents control, MD represents moderate drought, and SD represents severe drought. All the data in the table are analyzed by one-way ANOVA at *p* ≤ 0.05 and presented as mean ± SE (*n* = 5); the value in the same column is significantly different followed by different letters. Based on two-factor ANOVA, ** denotes highly a significant difference (*p* < 0.01), * denotes a significant difference (*p* < 0.05), and NS indicates no significant difference.

**Table 3:** Effects of spraying GA_3_ on nitrogen contents in plant and soil under drought.

Treatments		Aboveground Part of Plant (%)	Underground Part of Plant (%)	Total Content in Plant (%)	Absorbed from Soil (%)
CK	−GA_3_	31.936 ± 0.161 b	8.644 ± 0.112 b	40.580 ± 0.092 b	0.127 ± 0.003 a
	+GA_3_	36.418 ± 0.292 a	11.483 ± 0.007 a	47.900 ± 0.299 a	0.110 ± 0.000 a
MD	−GA_3_	24.481 ± 0.220 d	8.057 ± 0.056 c	32.537 ± 0.275 d	0.130 ± 0.010 a
	+GA_3_	29.240 ± 0.867 c	7.962 ± 0.034 c	37.202 ± 0.899 c	0.107 ± 0.003 a
SD	−GA_3_	23.643 ± 0.120 d	5.576 ± 0.027 e	29.219 ± 0.118 e	0.130 ± 0.010 a
	+GA_3_	24.759 ± 0.342 d	6.726 ± 0.014 d	31.485 ± 0.356 d	0.110 ± 0.015 a
Drought		**	**	**	NS
GA_3_		**	**	**	NS
Drought × GA_3_		*	**	**	NS

Note: +GA_3_ represents spraying with GA_3_; −GA_3_ represents not spraying GA_3_. CK represents control, MD represents moderate drought, and SD represents severe drought. All the data in the table are analyzed by one-way ANOVA at *p* ≤ 0.05 and presented as mean ± SE (*n* = 3); the value in the same column is significantly different followed by different letters. Based on two-factor ANOVA, ** denotes highly a significant difference (*p* < 0.01), * denotes a significant difference (*p* < 0.05), and NS indicates no significant difference.

**Table 4:** Effects of spraying GA_3_ on plant height and leaf area of plants under drought.

Growth Index	Treatments		Fifth Day Before Spraying GA_3_	Fifth Day After Spraying GA_3_
Plant height (cm)	CK	−GA_3_	67.40 ± 1.89 a	74.04 ± 1.43 a
		+GA_3_	67.64 ± 2.36 a	78.10 ± 3.58 a
	MD	−GA_3_	55.60 ± 2.62 b	63.34 ± 3.01 b
		+GA_3_	54.20 ± 0.80 b	69.44 ± 2.63 ab
	SD	−GA_3_	38.80 ± 4.32 c	44.60 ± 5.25 c
		+GA_3_	34.94 ± 0.93 c	41.32 ± 1.43 c
Leaf area (cm^2^)	CK	−GA_3_	39.59 ± 0.99 a	40.82 ± 0.97 a
		+GA_3_	40.58 ± 1.29 a	40.58 ± 0.95 a
	MD	−GA_3_	31.82 ± 1.02 b	31.86 ± 0.85 b
		+GA_3_	31.76 ± 1.20 b	31.50 ± 0.95 b
	SD	−GA_3_	16.46 ± 1.56 c	17.99 ± 2.31 c
		+GA_3_	16.67 ± 1.56 c	15.77 ± 1.22 c
Plant height	Drought		**	**
	GA_3_		NS	NS
	Drought × GA_3_		NS	NS
Leaf area	Drought		**	**
	GA_3_		NS	NS
	Drought × GA_3_		NS	NS

Note: +GA_3_ represents spraying with GA_3_; −GA_3_ represents without spraying GA_3_. CK represents control, MD represents moderate drought, and SD represents severe drought. All the data in the table are analyzed by one-way ANOVA at *p* ≤ 0.05 and presented as mean ± SE (*n* = 5); the value in the same column is significantly different followed by different letters. Based on two-factor ANOVA, ** denotes a highly significant difference (*p* < 0.01), and NS indicates no significant difference.

**Table 5:** Correlation analysis between parameters.

	Number of Pods	NUUE	NPT	NAT	IWUE	Plant Height	Leaf Area	*P*_N_	Total Nitrogen
ΔG_B_	−0.80 **	0.27	0.06	−0.39 *	0.13	0.37 *	0.30	0.24	−0.76 **
Number of pods		−0.21	0.07	0.29	−0.20	−0.03	−0.04	0.11	0.92 **
NUUE			−0.15	−0.41 *	0.46 *	0.38 *	0.44 *	0.42 *	0.11
NPT				0.15	−0.79 **	−0.34	−0.47 *	−0.43 *	−0.18
NAT					−0.22	−0.10	−0.07	−0.07	0.25
IWUE						0.47 **	0.57 **	0.52 **	0.08
Plant height							0.89 **	0.92 **	0.20
Leaf area								0.95 **	0.27
*P*_N_									0.37

Note: * means the correlation is significant at 0.05 level (two-tailed). ** means the correlation is significant at 0.01 level (double tail).

## Data Availability

The raw data supporting the conclusions of this article will be made available by the authors on request.
